# 1-(4-Bromo­phen­yl)-4,5-diphenyl-2-(1*H*-pyrrol-2-yl)-1*H*-imidazole

**DOI:** 10.1107/S2414314626001070

**Published:** 2026-02-03

**Authors:** Seeralan Nagaraj, Nagarajan Loganathan

**Affiliations:** a School of Chemistry Bharathidasan University, Tiruchirappalli 620 024 Tamilnadu India; b UGC Faculty Recharge Programme, New Delhi, India; Howard University, USA

**Keywords:** crystal structure, Debus–Radziszewski reaction, 1,2,4,5-tetra­substituted imidazoles, anion–π inter­actions, C—H⋯Br inter­actions

## Abstract

The title compound, C_25_H_18_BrN_3_, crystallizes in the triclinic *P*1 space group. It is of inter­est with respect to anti­cancer activity, anti­biotic, anti­bacterial and anti­fungal properties. The extended structure features N—H⋯N, C—H⋯N, C—H⋯π, N—H⋯π, C—H⋯Br and C—Br⋯π inter­actions.

## Abstract 2

A 1,2,4,5-tetra-substituted imidazole compound containing pyrole, 4-bromo­phenyl and phenyl groups has been synthesized in a four-component Debus–Radziszewski reaction of 1,2-diketone, aromatic aldehyde, aromatic amine and ammonium acetate

## Graphical abstract

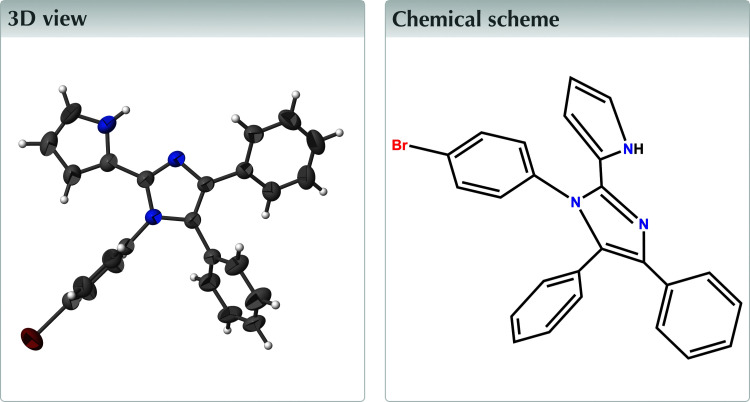

## Structure description

Imidazoles are one of the essential building blocks in many natural products and are of importance in the pharmaceutical industry. It is well known that the major constituent of most of the marine sponges contains bromo­pyrrole-imidazole alkaloids (Forte *et al.*, 2009 ▸; Lindel *et al.*, 2017 ▸; Zhang *et al.*, 2017 ▸). Several metalloenzymes consist of histidine (which contains an imidazole moiety) as one of the amino acids in their protein sequence. In addition, N-substituted imidazoles are vital ingredients in several known pharmacologically active metabolites, namely clotrimazole, ketoconazole, miconazole, oxicanozole (a well-known anti­biotic for the treatment of fungal infections), zoledronic acid (used for the treatment of osteoporosis), and nilotinib (an anti-cancer drug) (Yadav *et al.*, 2025 ▸). Similarly, several 1,2,4,5-tetra-substituted imidazole-based commercial drugs are available in the form of capravirine (anti-viral drug), losartan (angiotension receptor blocker), olmersatan, and medoximil (anti-hypertensive agent) (Gupta *et al.*, 2004 ▸; Narasimhan *et al.*, 2011 ▸; Siwach *et al.*, 2021 ▸).

Herein, we report the structure of a 1,2,4,5-tetra-substituted imidazole, namely, 1-(4-bromo­phen­yl)-4,5-diphenyl-2-(1*H*-pyrrol-2-yl)-1*H*-imidazole (**1**). To achieve this, many methods of synthesis were demonstrated (Zhang *et al.*, 2016 ▸; Hamdi *et al.*, 2024 ▸; Parameswari & Jayamoorthy, 2025 ▸). Among these, a multicomponent Debus–Radziszewski reaction involving benzil, pyrrole-2-carboxaldehyde, 4-bromo­aniline and ammonium acetate (1:1:3:3 ratio) in glacial acetic acid medium under overnight reflux condition afforded the title compound (**1**) as a white solid in very good yield (65–70%).

**Figure 1:**
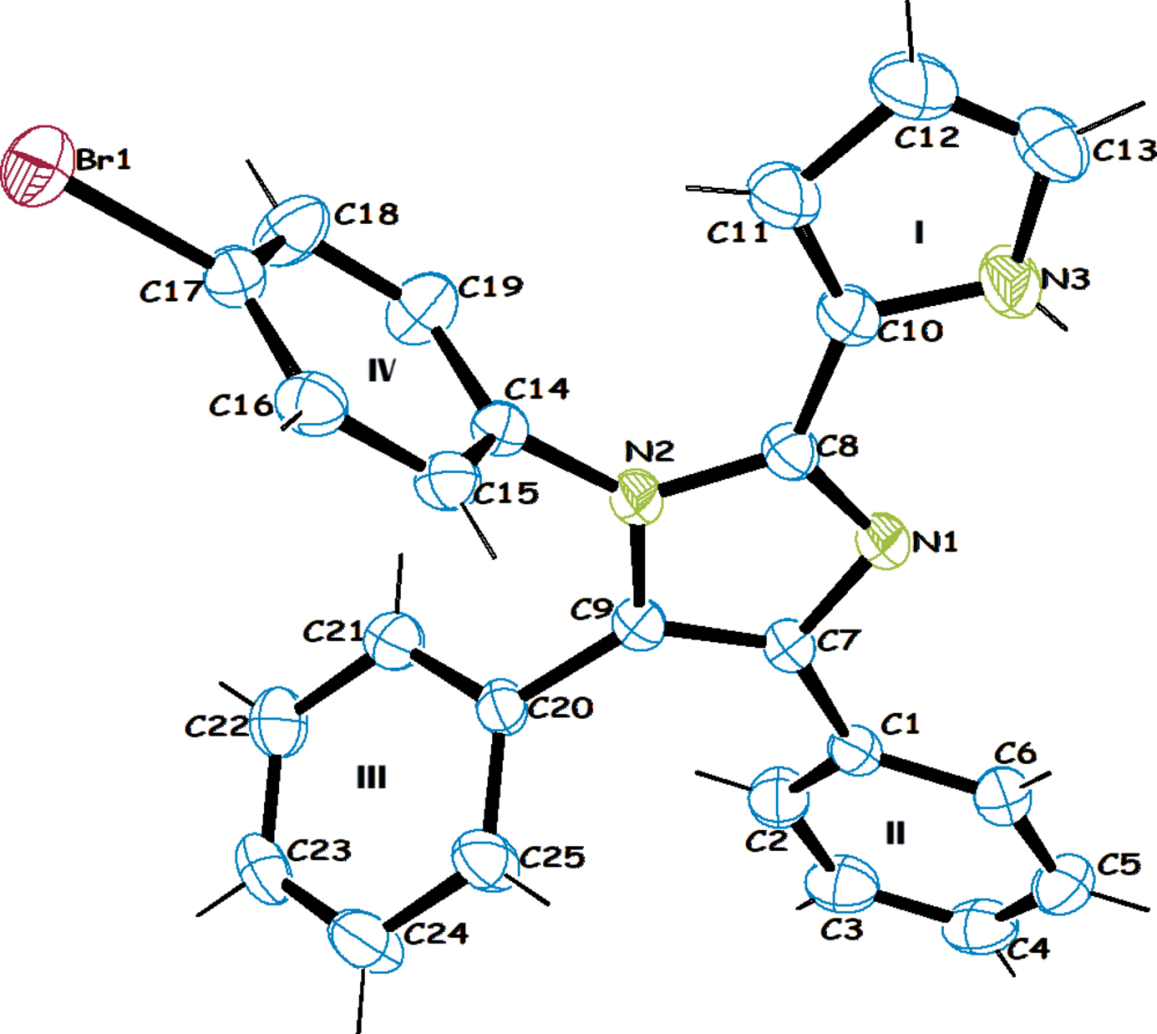
The mol­ecular structure of compound **1** with 50% probability displacement ellipsoids.

Compound **1** crystallizes in the triclinic *P*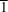 space group. Its bond parameters are in good agreement with those of previously determined 1,2,4,5-tetra-substituted imidazole derivatives (Gayathri *et al.*, 2010*a* ▸,*b* ▸,*c* ▸,*d* ▸; Xiao *et al.*, 2012 ▸; Zhao *et al.*, 2012 ▸). Fig. 1 ▸ shows the mol­ecular structure of **1** in which the four substituents on the imidazole are depicted as **I** to **IV**. The central imidazole ring is essentially coplanar with the 2-pyrrole ring [dihedral angle = 3.66 (18)]) while it is almost perpendicular to the 4-bromo­phenyl ring [88.6 (9)°]. The two phenyl rings attached in the 4- and 5-positions of the imidazole ring are not coplanar with it, subtending dihedral angles of 28.7 (9) and 63.3 (9)°, respectively. Intra­molecular C—H⋯N, N—H⋯N (Table 1 ▸) and C—H⋯π [C2—H2⋯π_Ph(II)_ 3.21Å and 134°] inter­actions occur.

**Figure 2:**
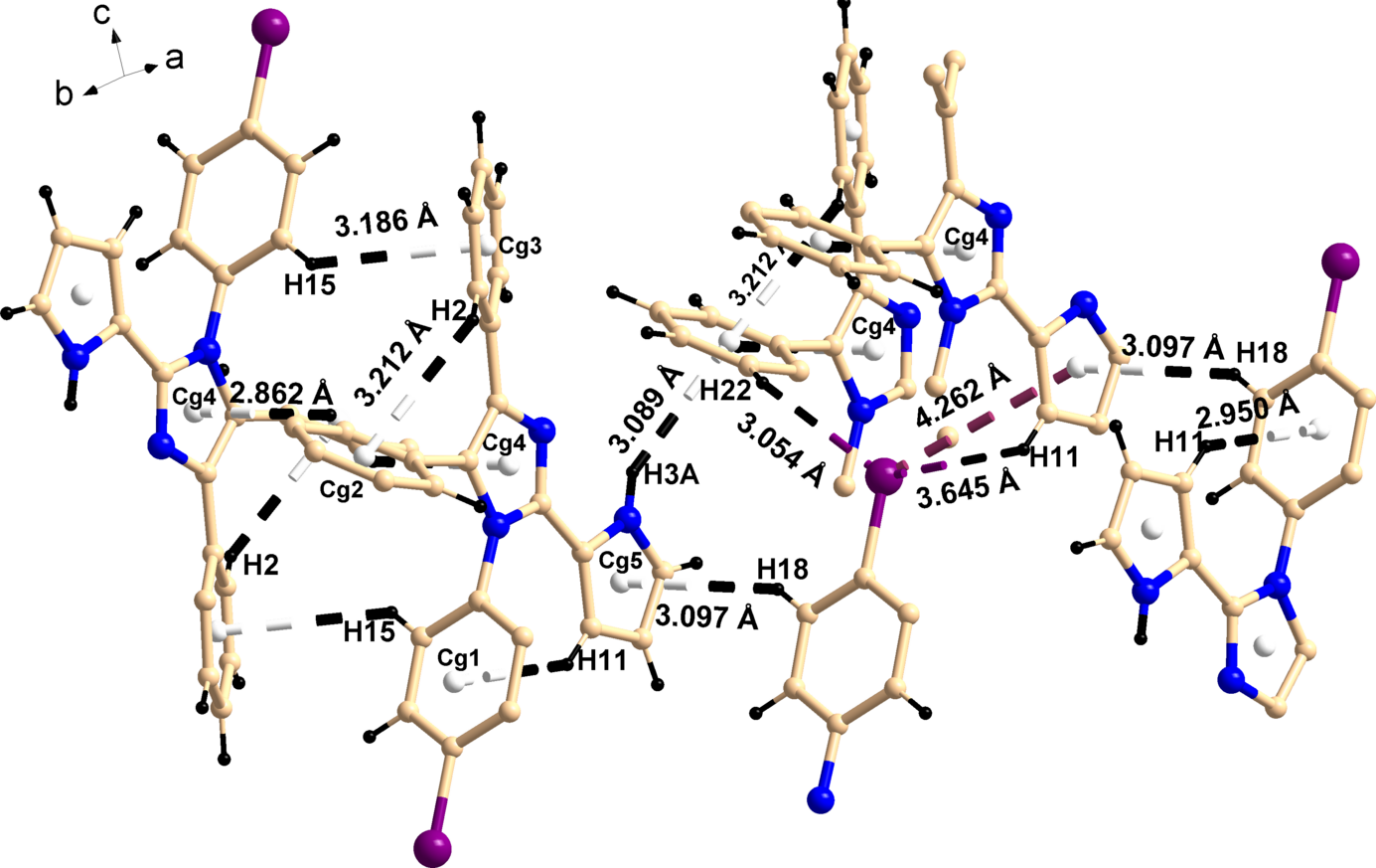
Perspective view of the C—H⋯π and C—H⋯Br inter­actions.

In the crystal, C—H⋯N (Table 1 ▸), C—H⋯π [C15—H15⋯π_Ph(II)_ 3.19 Å and 128°; symmetry code: 1 − *x*, 2 − *y*, 1 − *z*]; C18—H18⋯π_pyrrole_ 3.10 Å and 145°; symmetry code: 1 − *x*, 1 − *y*, −*z*; C25—H25⋯π_imidazole_ 2.86 Å and 136°; symmetry code: 1 − *x*, 2 − *y*, 1 − *z*] and N—H⋯π [N3—H3*A*⋯π_Ph(III)_ 3.09 Å and 148°; symmetry code: 1 + *x*, *y*, *z*] inter­actions are observed. Both intra­molecular and inter­molecular inter­actions are shown in (Fig. 2 ▸).

**Figure 3:**
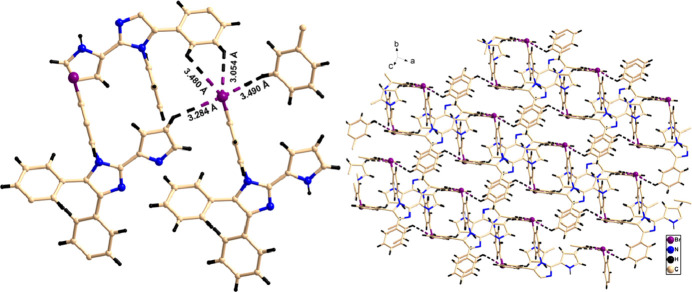
Perspective view of C—H⋯Br inter­actions and the supra­molecular architecture.

It is well established that weak C—H⋯halogen bonds (Desiraju *et al.*, 2005 ▸, 2011 ▸; Mazik *et al.*, 2010 ▸; Capdevila-Cortada *et al.*, 2015 ▸) and weak anion–π inter­actions (Schottel *et al.*, 2008 ▸) play a significant role in crystal engineering and supra­molecular chemistry. Herein, the bromine atom participates in various inter­molecular inter­actions *i.e.* C—Br⋯π inter­actions (C17—Br⋯π_pyrrole_ 4.26 Å and 122° symmetry code: 1 − *x*, 1 − *y*, −*z*) and C—H⋯Br inter­actions (C11—H11⋯Br1 3.64 Å 92°; symmetry code: *x*, −1 + *y*, *z*; C12—H12⋯Br1 3.288 Å 133°; symmetry code: −1 + *x*, *y*, *z*; C21—H21⋯Br1 3.48 Å and 119°; C22—H22⋯Br1 3.05 Å 139°; symmetry code: −*x*, 1 − *y*, −*z*) and eventually leading to the formation of a two-dimensional pillared network type supra­molecular architecture (Fig. 3 ▸).

## Synthesis and crystallization

A mixture of benzil (1.5324 g 7.28 mmol), 2-pyrrolecarbaldehyde (0.6847 g, 7.2 mmol), 4-bromo­aniline (4.9602 g, 28.8 mmol) and ammonium acetate (3.7555 g, 28.8 mmol) was dissolved in 35 ml of glacial acetic acid and the mixture was allowed to reflux overnight. The reaction was monitored by TLC; after completion, the reaction was quenched by pouring the solution in to a crushed ice bath, the obtained white precipitate was filtered and purified by column chromatography using silica gel and hexane and ethyl­acetate (9:1) as eluents. Yield 65–70%, m.p. 232°C. FT–IR (cm^−1^) 1010(*s*), 1093(*w*), 1281(*w*), 1489(*w*), 1587(*w*), 3029(*w*), 3204 (*br*).

## Refinement

Crystal data, data collection and structure refinement details are summarized in Table 2 ▸.

## Supplementary Material

Crystal structure: contains datablock(s) I. DOI: 10.1107/S2414314626001070/bv4059sup1.cif

Structure factors: contains datablock(s) I. DOI: 10.1107/S2414314626001070/bv4059Isup2.hkl

Supporting information file. DOI: 10.1107/S2414314626001070/bv4059Isup3.cml

CCDC reference: 2224025

Additional supporting information:  crystallographic information; 3D view; checkCIF report

## Figures and Tables

**Table 1:** Hydrogen-bond geometry (Å, °)

*D*—H⋯*A*	*D*—H	H⋯*A*	*D*⋯*A*	*D*—H⋯*A*
N3—H3*A*⋯N1	0.86	2.52	2.774 (3)	98
C6—H6⋯N1	0.93	2.61	2.893 (3)	98
C23—H23⋯N1^i^	0.93	2.78	3.470 (3)	132
C24—H24⋯N1^ii^	0.93	2.98	3.547 (4)	121

Symmetry codes: (i) 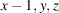; (ii) 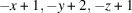.

**Table 2:** Experimental details

Crystal data	
Chemical formula	C_25_H_18_BrN_3_
*M*_r_	440.33
Crystal system, space group	Triclinic, *P* 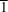
Temperature (K)	300
*a*, *b*, *c* (Å)	9.6044 (9), 9.7662 (7), 12.3705 (11)
α, β, γ (°)	103.837 (3), 92.480 (5), 113.592 (2)
*V* (Å^3^)	1019.82 (15)
*Z*	2
Radiation type	Mo *K*α
μ (mm^−1^)	2.03
Crystal size (mm)	0.29 × 0.20 × 0.17

Data collection	
Diffractometer	Bruker D8 QUEST diffractometer with PHOTON II detector
Absorption correction	Multi-scan (*SADABS*; Krause *et al.*, 2015 ▸)
*T*_min_, *T*_max_	0.590, 0.724
No. of measured, independent and observed [*I* > 2σ(*I*)] reflections	28057, 5046, 3328
*R*_int_	0.044
(sin θ/λ)_max_ (Å^−1^)	0.667

Refinement	
*R*[*F*^2^ > 2σ(*F*^2^)], *wR*(*F*^2^), *S*	0.047, 0.111, 1.02
No. of reflections	5046
No. of parameters	262
H-atom treatment	H-atom parameters constrained
Δρ_max_, Δρ_min_ (e Å^−3^)	0.70, −0.67

Computer programs: *APEX4* and *SAINT* (Bruker, 2012 ▸), *SHELXT* (Sheldrick, 2015*a* ▸), *SHELXL2019/2* (Sheldrick, 2015*b* ▸), *ORTEP-3 for Windows* (Farrugia, 2012 ▸), *DIAMOND* (Brandenburg *et al.*, 2014 ▸) and *publCIF* (Westrip, 2010 ▸).

## full crystallographic data

### 1-(4-Bromophenyl)-4,5-diphenyl-2-(1H-pyrrol-2-yl)-1H-imidazole Crystal data

**Table:**

C_25_H_18_BrN_3_	*Z* = 2
*M**_r_* = 440.33	*F*(000) = 448
Triclinic, *P*1	*D*_x_ = 1.434 Mg m^−^^3^
*a* = 9.6044 (9) Å	Mo *K*α radiation, λ = 0.71073 Å
*b* = 9.7662 (7) Å	Cell parameters from 9931 reflections
*c* = 12.3705 (11) Å	θ = 2.3–23.5°
α = 103.837 (3)°	µ = 2.03 mm^−^^1^
β = 92.480 (5)°	*T* = 300 K
γ = 113.592 (2)°	Block, colourless
*V* = 1019.82 (15) Å^3^	0.29 × 0.20 × 0.17 mm

### 1-(4-Bromophenyl)-4,5-diphenyl-2-(1H-pyrrol-2-yl)-1H-imidazole Data collection

**Table:**

Bruker D8 QUEST diffractometer with PHOTON II detector	*R*_int_ = 0.044
Radiation source: i-mu-s microfocus source	θ_max_ = 28.3°, θ_min_ = 1.7°
φ and ω scans	*h* = −12→12
Absorption correction: multi-scan (SADABS; Krause *et al.*, 2015)	*k* = −13→13
*T*_min_ = 0.590, *T*_max_ = 0.724	*l* = −16→16
28057 measured reflections	4 standard reflections every 19 reflections
5046 independent reflections	intensity decay: none
3328 reflections with *I* > 2σ(*I*)	

### 1-(4-Bromophenyl)-4,5-diphenyl-2-(1H-pyrrol-2-yl)-1H-imidazole Refinement

**Table:**

Refinement on *F*^2^	0 restraints
Least-squares matrix: full	Hydrogen site location: inferred from neighbouring sites
*R*[*F*^2^ > 2σ(*F*^2^)] = 0.047	H-atom parameters constrained
*wR*(*F*^2^) = 0.111	*w* = 1/[σ^2^(*F*_o_^2^) + (0.0391*P*)^2^ + 0.6237*P*] where *P* = (*F*_o_^2^ + 2*F*_c_^2^)/3
*S* = 1.02	(Δ/σ)_max_ = 0.001
5046 reflections	Δρ_max_ = 0.70 e Å^−^^3^
262 parameters	Δρ_min_ = −0.67 e Å^−^^3^

### 1-(4-Bromophenyl)-4,5-diphenyl-2-(1H-pyrrol-2-yl)-1H-imidazole Special details

**Table:**

Geometry. All esds (except the esd in the dihedral angle between two l.s. planes) are estimated using the full covariance matrix. The cell esds are taken into account individually in the estimation of esds in distances, angles and torsion angles; correlations between esds in cell parameters are only used when they are defined by crystal symmetry. An approximate (isotropic) treatment of cell esds is used for estimating esds involving l.s. planes.
Refinement. All the non-hydrogen atoms were refined anisotropically using full-matrix least-square procedures while the hydrogen atoms were included in the idealized position and N—H proton was added from the difference Fourier map. C—H bonds were constrained to 0.95 Å for aromatic C—H (with *U*_iso_(H) of 1.2).

### 1-(4-Bromophenyl)-4,5-diphenyl-2-(1H-pyrrol-2-yl)-1H-imidazole Fractional atomic coordinates and isotropic or equivalent isotropic displacement parameters (Å^2^)

**Table:**

	*x*	*y*	*z*	*U*_iso_*/*U*_eq_	
Br1	0.17363 (4)	0.77956 (4)	−0.13312 (3)	0.07507 (15)	
N1	0.6881 (2)	0.7667 (2)	0.40296 (17)	0.0435 (5)	
N2	0.4941 (2)	0.7513 (2)	0.28907 (16)	0.0424 (5)	
N3	0.8849 (3)	0.7915 (3)	0.2437 (2)	0.0631 (7)	
H3A	0.919538	0.786406	0.307202	0.076*	
C1	0.5738 (3)	0.7303 (3)	0.5743 (2)	0.0424 (5)	
C2	0.4476 (3)	0.6416 (3)	0.6162 (2)	0.0542 (7)	
H2	0.351403	0.591522	0.571249	0.065*	
C3	0.4624 (4)	0.6265 (4)	0.7230 (2)	0.0629 (8)	
H3	0.376028	0.567672	0.750074	0.076*	
C4	0.6027 (4)	0.6970 (4)	0.7900 (2)	0.0664 (9)	
H4	0.612151	0.685934	0.862259	0.080*	
C5	0.7299 (4)	0.7845 (4)	0.7499 (2)	0.0689 (9)	
H5	0.825907	0.831789	0.794895	0.083*	
C6	0.7161 (3)	0.8026 (3)	0.6430 (2)	0.0553 (7)	
H6	0.802551	0.863354	0.616948	0.066*	
C7	0.5633 (3)	0.7454 (3)	0.45918 (19)	0.0403 (5)	
C8	0.6437 (3)	0.7692 (3)	0.3020 (2)	0.0419 (5)	
C9	0.4418 (3)	0.7368 (3)	0.39117 (19)	0.0399 (5)	
C10	0.7427 (3)	0.7854 (3)	0.2166 (2)	0.0464 (6)	
C11	0.7341 (4)	0.7976 (4)	0.1089 (2)	0.0619 (8)	
H11	0.650583	0.797226	0.067589	0.074*	
C12	0.8737 (4)	0.8109 (4)	0.0719 (3)	0.0693 (9)	
H12	0.899548	0.820796	0.001732	0.083*	
C13	0.9628 (4)	0.8067 (4)	0.1560 (3)	0.0706 (9)	
H13	1.061884	0.813235	0.154215	0.085*	
C14	0.4130 (3)	0.7570 (3)	0.19131 (19)	0.0406 (5)	
C15	0.4253 (3)	0.8973 (3)	0.1795 (2)	0.0466 (6)	
H15	0.481108	0.988158	0.237019	0.056*	
C16	0.3550 (3)	0.9038 (3)	0.0824 (2)	0.0501 (6)	
H16	0.364313	0.998937	0.073592	0.060*	
C17	0.2714 (3)	0.7691 (3)	−0.0007 (2)	0.0478 (6)	
C18	0.2563 (4)	0.6277 (3)	0.0102 (2)	0.0623 (8)	
H18	0.199101	0.536796	−0.046879	0.075*	
C19	0.3278 (4)	0.6230 (3)	0.1077 (2)	0.0596 (7)	
H19	0.317994	0.527791	0.116641	0.071*	
C20	0.2879 (3)	0.7227 (3)	0.41275 (19)	0.0404 (5)	
C21	0.1557 (3)	0.5947 (3)	0.3567 (2)	0.0592 (7)	
H21	0.161990	0.516775	0.299562	0.071*	
C22	0.0139 (3)	0.5807 (4)	0.3844 (3)	0.0688 (8)	
H22	−0.074278	0.493345	0.345820	0.083*	
C23	0.0017 (3)	0.6925 (4)	0.4671 (3)	0.0624 (8)	
H23	−0.094174	0.681746	0.485665	0.075*	
C24	0.1309 (4)	0.8209 (4)	0.5229 (3)	0.0706 (9)	
H24	0.123314	0.898620	0.579398	0.085*	
C25	0.2730 (3)	0.8356 (3)	0.4957 (3)	0.0594 (7)	
H25	0.360480	0.923780	0.534372	0.071*	

### 1-(4-Bromophenyl)-4,5-diphenyl-2-(1H-pyrrol-2-yl)-1H-imidazole Atomic displacement parameters (Å^2^)

**Table:**

	*U*^11^	*U*^22^	*U*^33^	*U*^12^	*U*^13^	*U*^23^
Br1	0.0872 (3)	0.0988 (3)	0.0531 (2)	0.0534 (2)	0.00014 (16)	0.02267 (17)
N1	0.0363 (11)	0.0534 (12)	0.0419 (11)	0.0208 (10)	0.0091 (9)	0.0118 (9)
N2	0.0361 (11)	0.0536 (12)	0.0395 (10)	0.0209 (9)	0.0080 (8)	0.0129 (9)
N3	0.0405 (13)	0.0945 (19)	0.0583 (14)	0.0273 (13)	0.0178 (11)	0.0288 (13)
C1	0.0450 (14)	0.0476 (14)	0.0398 (12)	0.0263 (12)	0.0085 (11)	0.0093 (11)
C2	0.0542 (16)	0.0591 (17)	0.0507 (15)	0.0235 (14)	0.0106 (13)	0.0183 (13)
C3	0.084 (2)	0.0631 (18)	0.0529 (17)	0.0374 (17)	0.0238 (16)	0.0227 (14)
C4	0.105 (3)	0.072 (2)	0.0411 (15)	0.056 (2)	0.0121 (17)	0.0175 (14)
C5	0.076 (2)	0.085 (2)	0.0468 (16)	0.0462 (19)	−0.0089 (15)	0.0020 (15)
C6	0.0500 (16)	0.0701 (18)	0.0473 (15)	0.0307 (14)	0.0055 (12)	0.0099 (13)
C7	0.0353 (13)	0.0457 (14)	0.0398 (12)	0.0184 (11)	0.0071 (10)	0.0092 (10)
C8	0.0380 (13)	0.0442 (13)	0.0435 (13)	0.0176 (11)	0.0101 (11)	0.0114 (11)
C9	0.0372 (13)	0.0433 (13)	0.0388 (12)	0.0174 (11)	0.0077 (10)	0.0100 (10)
C10	0.0437 (14)	0.0490 (15)	0.0480 (14)	0.0199 (12)	0.0135 (11)	0.0147 (11)
C11	0.0630 (18)	0.085 (2)	0.0520 (16)	0.0407 (17)	0.0222 (14)	0.0252 (15)
C12	0.073 (2)	0.082 (2)	0.0587 (18)	0.0331 (18)	0.0336 (17)	0.0251 (16)
C13	0.0494 (18)	0.086 (2)	0.076 (2)	0.0257 (16)	0.0306 (16)	0.0241 (18)
C14	0.0391 (13)	0.0471 (14)	0.0375 (12)	0.0202 (11)	0.0094 (10)	0.0114 (10)
C15	0.0478 (15)	0.0439 (14)	0.0440 (13)	0.0194 (12)	0.0072 (11)	0.0051 (11)
C16	0.0609 (17)	0.0471 (15)	0.0506 (15)	0.0297 (13)	0.0138 (13)	0.0154 (12)
C17	0.0495 (15)	0.0612 (17)	0.0406 (13)	0.0299 (13)	0.0095 (11)	0.0160 (12)
C18	0.078 (2)	0.0446 (16)	0.0490 (16)	0.0191 (14)	−0.0093 (14)	0.0020 (12)
C19	0.075 (2)	0.0434 (15)	0.0551 (16)	0.0234 (14)	−0.0053 (14)	0.0114 (13)
C20	0.0356 (13)	0.0465 (14)	0.0417 (12)	0.0190 (11)	0.0073 (10)	0.0142 (11)
C21	0.0454 (16)	0.0628 (18)	0.0566 (16)	0.0206 (14)	0.0049 (13)	−0.0014 (13)
C22	0.0341 (15)	0.076 (2)	0.078 (2)	0.0147 (14)	0.0019 (14)	0.0055 (17)
C23	0.0400 (16)	0.080 (2)	0.079 (2)	0.0341 (16)	0.0190 (14)	0.0265 (17)
C24	0.0574 (19)	0.0584 (18)	0.094 (2)	0.0293 (16)	0.0272 (17)	0.0063 (17)
C25	0.0427 (15)	0.0496 (16)	0.0750 (19)	0.0157 (13)	0.0168 (14)	0.0037 (14)

### 1-(4-Bromophenyl)-4,5-diphenyl-2-(1H-pyrrol-2-yl)-1H-imidazole Geometric parameters (Å, º)

**Table:**

Br1—C17	1.897 (2)	C11—H11	0.9300
N1—C8	1.312 (3)	C12—C13	1.336 (5)
N1—C7	1.381 (3)	C12—H12	0.9300
N2—C8	1.373 (3)	C13—H13	0.9300
N2—C9	1.397 (3)	C14—C19	1.369 (4)
N2—C14	1.434 (3)	C14—C15	1.371 (3)
N3—C13	1.348 (4)	C15—C16	1.380 (4)
N3—C10	1.366 (3)	C15—H15	0.9300
N3—H3A	0.8600	C16—C17	1.367 (4)
C1—C2	1.385 (4)	C16—H16	0.9300
C1—C6	1.389 (4)	C17—C18	1.370 (4)
C1—C7	1.469 (3)	C18—C19	1.382 (4)
C2—C3	1.372 (4)	C18—H18	0.9300
C2—H2	0.9300	C19—H19	0.9300
C3—C4	1.366 (5)	C20—C25	1.374 (4)
C3—H3	0.9300	C20—C21	1.377 (4)
C4—C5	1.374 (5)	C21—C22	1.380 (4)
C4—H4	0.9300	C21—H21	0.9300
C5—C6	1.383 (4)	C22—C23	1.353 (4)
C5—H5	0.9300	C22—H22	0.9300
C6—H6	0.9300	C23—C24	1.362 (4)
C7—C9	1.370 (3)	C23—H23	0.9300
C8—C10	1.446 (3)	C24—C25	1.379 (4)
C9—C20	1.471 (3)	C24—H24	0.9300
C10—C11	1.368 (4)	C25—H25	0.9300
C11—C12	1.402 (4)		
			
C8—N1—C7	106.17 (19)	C13—C12—H12	126.2
C8—N2—C9	106.67 (19)	C11—C12—H12	126.2
C8—N2—C14	125.72 (19)	C12—C13—N3	108.6 (3)
C9—N2—C14	127.50 (19)	C12—C13—H13	125.7
C13—N3—C10	109.8 (3)	N3—C13—H13	125.7
C13—N3—H3A	125.1	C19—C14—C15	119.9 (2)
C10—N3—H3A	125.1	C19—C14—N2	120.0 (2)
C2—C1—C6	118.2 (2)	C15—C14—N2	120.1 (2)
C2—C1—C7	122.4 (2)	C14—C15—C16	120.1 (2)
C6—C1—C7	119.3 (2)	C14—C15—H15	120.0
C3—C2—C1	120.9 (3)	C16—C15—H15	120.0
C3—C2—H2	119.6	C17—C16—C15	119.3 (2)
C1—C2—H2	119.6	C17—C16—H16	120.3
C4—C3—C2	120.7 (3)	C15—C16—H16	120.3
C4—C3—H3	119.7	C16—C17—C18	121.4 (2)
C2—C3—H3	119.7	C16—C17—Br1	118.9 (2)
C3—C4—C5	119.4 (3)	C18—C17—Br1	119.7 (2)
C3—C4—H4	120.3	C17—C18—C19	118.7 (2)
C5—C4—H4	120.3	C17—C18—H18	120.7
C4—C5—C6	120.4 (3)	C19—C18—H18	120.7
C4—C5—H5	119.8	C14—C19—C18	120.6 (2)
C6—C5—H5	119.8	C14—C19—H19	119.7
C5—C6—C1	120.3 (3)	C18—C19—H19	119.7
C5—C6—H6	119.9	C25—C20—C21	117.7 (2)
C1—C6—H6	119.9	C25—C20—C9	120.0 (2)
C9—C7—N1	110.3 (2)	C21—C20—C9	122.3 (2)
C9—C7—C1	130.0 (2)	C20—C21—C22	120.7 (3)
N1—C7—C1	119.7 (2)	C20—C21—H21	119.7
N1—C8—N2	111.5 (2)	C22—C21—H21	119.7
N1—C8—C10	122.5 (2)	C23—C22—C21	120.8 (3)
N2—C8—C10	126.0 (2)	C23—C22—H22	119.6
C7—C9—N2	105.3 (2)	C21—C22—H22	119.6
C7—C9—C20	131.4 (2)	C22—C23—C24	119.5 (3)
N2—C9—C20	123.2 (2)	C22—C23—H23	120.2
N3—C10—C11	106.4 (2)	C24—C23—H23	120.2
N3—C10—C8	117.0 (2)	C23—C24—C25	120.0 (3)
C11—C10—C8	136.6 (3)	C23—C24—H24	120.0
C10—C11—C12	107.7 (3)	C25—C24—H24	120.0
C10—C11—H11	126.2	C20—C25—C24	121.3 (3)
C12—C11—H11	126.2	C20—C25—H25	119.3
C13—C12—C11	107.5 (3)	C24—C25—H25	119.3
			
C6—C1—C2—C3	0.6 (4)	N2—C8—C10—C11	4.6 (5)
C7—C1—C2—C3	178.1 (2)	N3—C10—C11—C12	0.1 (3)
C1—C2—C3—C4	−1.0 (4)	C8—C10—C11—C12	179.9 (3)
C2—C3—C4—C5	0.3 (4)	C10—C11—C12—C13	−0.1 (4)
C3—C4—C5—C6	0.7 (5)	C11—C12—C13—N3	0.0 (4)
C4—C5—C6—C1	−1.1 (4)	C10—N3—C13—C12	0.1 (4)
C2—C1—C6—C5	0.4 (4)	C8—N2—C14—C19	92.0 (3)
C7—C1—C6—C5	−177.2 (2)	C9—N2—C14—C19	−92.4 (3)
C8—N1—C7—C9	−0.7 (3)	C8—N2—C14—C15	−85.6 (3)
C8—N1—C7—C1	177.3 (2)	C9—N2—C14—C15	90.0 (3)
C2—C1—C7—C9	28.5 (4)	C19—C14—C15—C16	−1.6 (4)
C6—C1—C7—C9	−154.0 (3)	N2—C14—C15—C16	176.1 (2)
C2—C1—C7—N1	−149.1 (2)	C14—C15—C16—C17	1.0 (4)
C6—C1—C7—N1	28.4 (3)	C15—C16—C17—C18	−0.2 (4)
C7—N1—C8—N2	0.4 (3)	C15—C16—C17—Br1	179.37 (19)
C7—N1—C8—C10	−178.3 (2)	C16—C17—C18—C19	−0.1 (4)
C9—N2—C8—N1	0.1 (3)	Br1—C17—C18—C19	−179.6 (2)
C14—N2—C8—N1	176.5 (2)	C15—C14—C19—C18	1.3 (4)
C9—N2—C8—C10	178.7 (2)	N2—C14—C19—C18	−176.3 (3)
C14—N2—C8—C10	−4.9 (4)	C17—C18—C19—C14	−0.5 (5)
N1—C7—C9—N2	0.8 (3)	C7—C9—C20—C25	59.9 (4)
C1—C7—C9—N2	−177.0 (2)	N2—C9—C20—C25	−117.1 (3)
N1—C7—C9—C20	−176.5 (2)	C7—C9—C20—C21	−116.3 (3)
C1—C7—C9—C20	5.7 (4)	N2—C9—C20—C21	66.8 (3)
C8—N2—C9—C7	−0.6 (3)	C25—C20—C21—C22	−0.6 (4)
C14—N2—C9—C7	−176.8 (2)	C9—C20—C21—C22	175.6 (3)
C8—N2—C9—C20	177.1 (2)	C20—C21—C22—C23	0.1 (5)
C14—N2—C9—C20	0.8 (4)	C21—C22—C23—C24	0.5 (5)
C13—N3—C10—C11	−0.1 (3)	C22—C23—C24—C25	−0.6 (5)
C13—N3—C10—C8	−179.9 (2)	C21—C20—C25—C24	0.6 (4)
N1—C8—C10—N3	2.7 (4)	C9—C20—C25—C24	−175.7 (3)
N2—C8—C10—N3	−175.7 (2)	C23—C24—C25—C20	0.0 (5)
N1—C8—C10—C11	−177.0 (3)		

### 1-(4-Bromophenyl)-4,5-diphenyl-2-(1H-pyrrol-2-yl)-1H-imidazole Hydrogen-bond geometry (Å, º)

**Table:**

*D*—H···*A*	*D*—H	H···*A*	*D*···*A*	*D*—H···*A*
N3—H3*A*···N1	0.86	2.52	2.774 (3)	98
C6—H6···N1	0.93	2.61	2.893 (3)	98
C23—H23···N1^i^	0.93	2.78	3.470 (3)	132
C24—H24···N1^ii^	0.93	2.98	3.547 (4)	121

Symmetry codes: (i) *x*−1, *y*, *z*; (ii) −*x*+1, −*y*+2, −*z*+1.
